# Periodontal Endoscopy-Assisted Minimally Invasive Nonsurgical Therapy Versus Regenerative Surgery for the Treatment of Intrabony Defects: A Narrative Review

**DOI:** 10.3390/healthcare14080977

**Published:** 2026-04-08

**Authors:** Sylwia Jakubowska, Jan Kowalski

**Affiliations:** Department of Periodontology and Oral Mucosa Diseases, Medical University of Warsaw, Binieckiego 6 St., 02-097 Warsaw, Poland; jkowalski@wum.edu.pl

**Keywords:** periodontal endoscope, perioscope, periodontal microsurgery, periodontal regeneration, periodontitis, intrabony defects

## Abstract

**Background:** The aim of the present narrative review is to synthesize the available scientific evidence comparing periodontal endoscopy-assisted therapy with established surgical regenerative procedures for the treatment of intrabony periodontal defects. While regenerative surgery—including papilla-preserving techniques—remains the standard approach for achieving predictable clinical attachment gain, these procedures may potentially compromise papillary integrity and healing dynamics. Periodontal endoscopy enables enhanced visualization and debridement without surgical access. This review evaluates available studies and discusses whether endoscopy-assisted therapy can achieve outcomes comparable to surgical regeneration while reducing tissue trauma and preserving interdental anatomy. **Methods:** A comprehensive literature search was conducted using the electronic databases PubMed, Web of Science, The Cochrane Library, and Scopus, supplemented by manual searching. The search was performed up to 1 November 2025. **Results:** Two studies were included. Overall, there is a substantial lack of RCTs directly comparing periodontal endoscopy-assisted therapy with surgical regenerative procedures. However, EASD (Endoscopic- assisted subgingival debridement) was found not to be inferior to papilla-preservation surgery (PPFS) for treating residual pockets in intrabony defects. Both PPFS and EASD were effective, although PPFS showed more consistent microbial modulation. **Conclusions:** Periodontal endoscopy-assisted therapy may be considered a promising minimally invasive approach for selected intrabony defects, potentially reducing surgical morbidity and preserving interdental tissues. Although early data suggest that endoscopy-guided approaches may offer comparable clinical improvements with less invasiveness, the evidence base is too small to support definitive recommendations. Robust, well-designed randomized trials are needed to define its clinical indications and compare it directly with established regenerative procedures.

## 1. Introduction

The main objective of periodontal intervention is to suppress the inflammatory process. The therapeutic protocol requires the mechanical debridement (removal) of the subgingival microbial biofilm. This critical procedural step is necessary for the re-establishment of a localized, physiologically compatible microenvironment that promotes the long-term stability and health of the periodontium [1]. The difficulty in achieving effective subgingival debridement represents one of the key factors underlying the failure to achieve pocket resolution—defined as the absence of pockets > 5 mm and the absence of pockets > 4 mm exhibiting bleeding on probing (BOP) [2,3]. This challenge is particularly evident in deep periodontal pockets, those associated with intrabony defects, and in sites with complex root morphologies such as concavities and furcation involvements [4,5]. Periodontal regenerative surgery is performed when conventional non-surgical therapy fails to achieve disease resolution in sites with advanced attachment loss, particularly those associated with deep intrabony defects. The primary rationale for regenerative intervention is not merely pocket reduction, but the reconstruction of the lost periodontal supporting apparatus—including alveolar bone, periodontal ligament, and cementum—in order to re-establish a functional attachment and improve the long-term prognosis of compromised teeth. Treatment of intrabony defects has been extensively investigated, with numerous randomized controlled clinical trials and long-term studies addressing this topic [6,7,8,9,10,11,12]. When such defects remain untreated, they commonly exhibit progressive deterioration, and the risk of tooth loss is significantly greater compared with sites exhibiting a healthy periodontium [13]. A variety of therapeutic strategies have been introduced for managing intrabony defects, including open flap debridement, scaling and root planing (SRP), and periodontal regenerative approaches [14]. Notably, regenerative procedures have consistently demonstrated superior outcomes compared with open flap surgery in the treatment of these defects [15,16]. Nevertheless, despite substantial advancements in surgical techniques, gingival recession and postoperative discomfort continue to pose challenges for both patients and clinicians [17,18].

In this context, periodontal endoscopy-assisted subgingival debridement has emerged as a minimally invasive approach aimed at improving visualization and debridement without flap elevation. However, its clinical effectiveness relative to established surgical regenerative procedures for intrabony defects remains unclear.

### 1.1. The Evolution of Periodontal Regeneration: Transitioning to Biologics and Minimal Flap Surgery

The foundation of periodontal regeneration is achieving pocket closure without significant gingival recession, enhancing the clinical attachment level to improve the prognosis of compromised teeth, and ensuring long-term periodontal stability [19,20,21,22]. The focus moved away from placing barrier membranes (used in older Guided Tissue Regeneration, or GTR, techniques) to using biologics [23]. Clinicians adopted sophisticated agents like Recombinant Growth Factors, Amelogenins (e.g., Emdogain), and Hyaluronic Acid. This change resulted in a significantly higher rate of primary wound closure and a lower rate of wound breakdown (wound failure), largely because these biologics facilitate healing and require less tension for flap closure [24,25]. Improvements in visualization and instrumentation allowed surgeons to handle tissue more delicately. New microsurgical techniques led to improved flap revascularization (better blood flow to the flap) and a superior primary wound closure rate, crucial factors for predictable healing [6,26]. While the application of biomaterials is not necessarily essential and has become of secondary importance, establishing an optimal local environment to exploit the inherent regenerative potential of periodontal tissues remains the main focus [27,28,29,30]. Despite its recorded successes, regenerative periodontal surgery is problematic due to patient morbidity, potential complications, and high material costs, with outcomes frequently being unpredictable and characterized by reports of clinical failures and incomplete therapeutic success.

### 1.2. Minimally Invasive and Papilla Preservation Techniques

**Figure 1:**
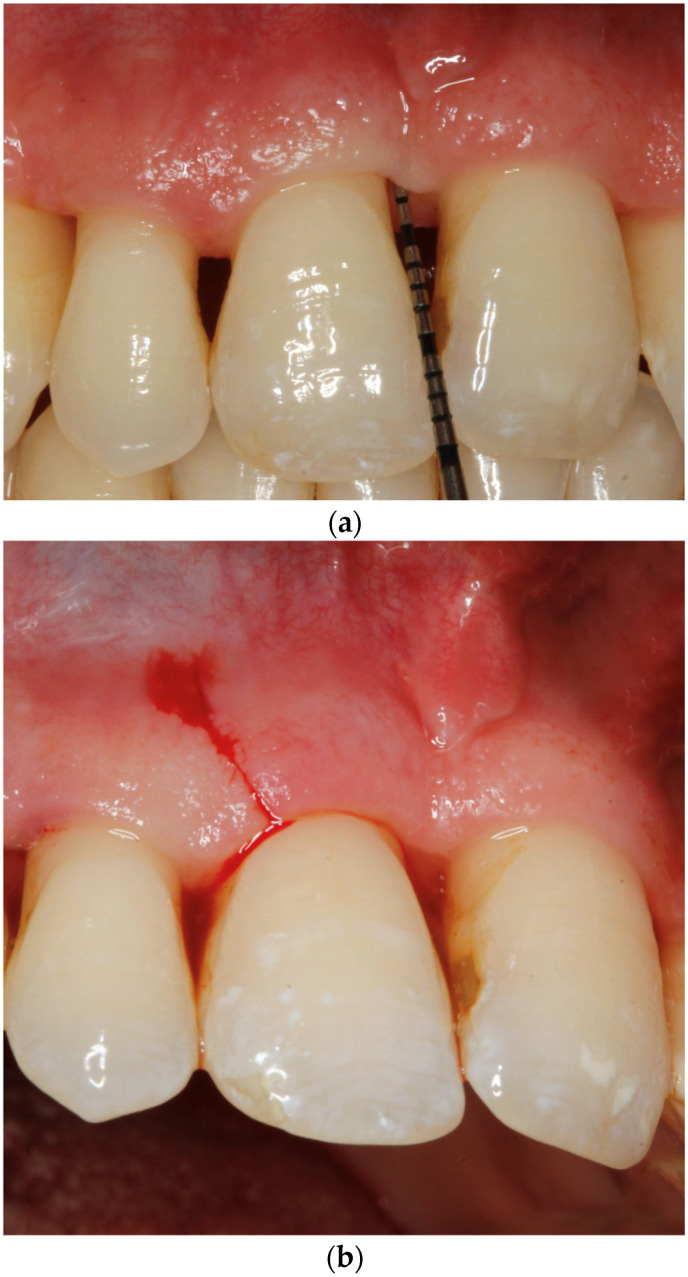

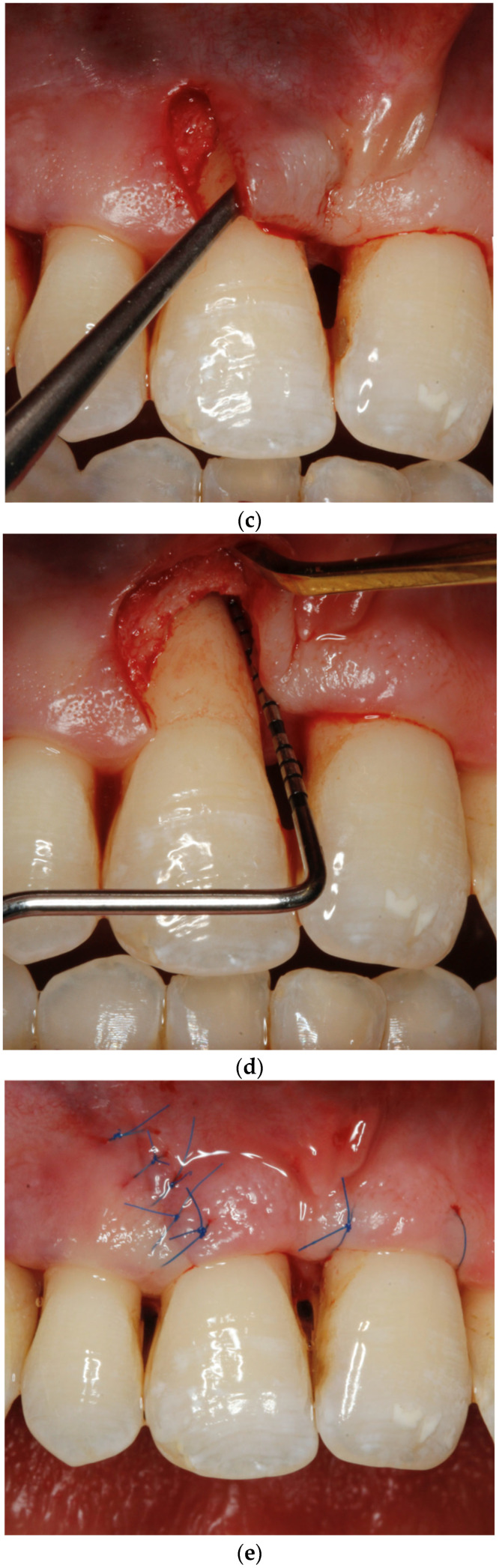

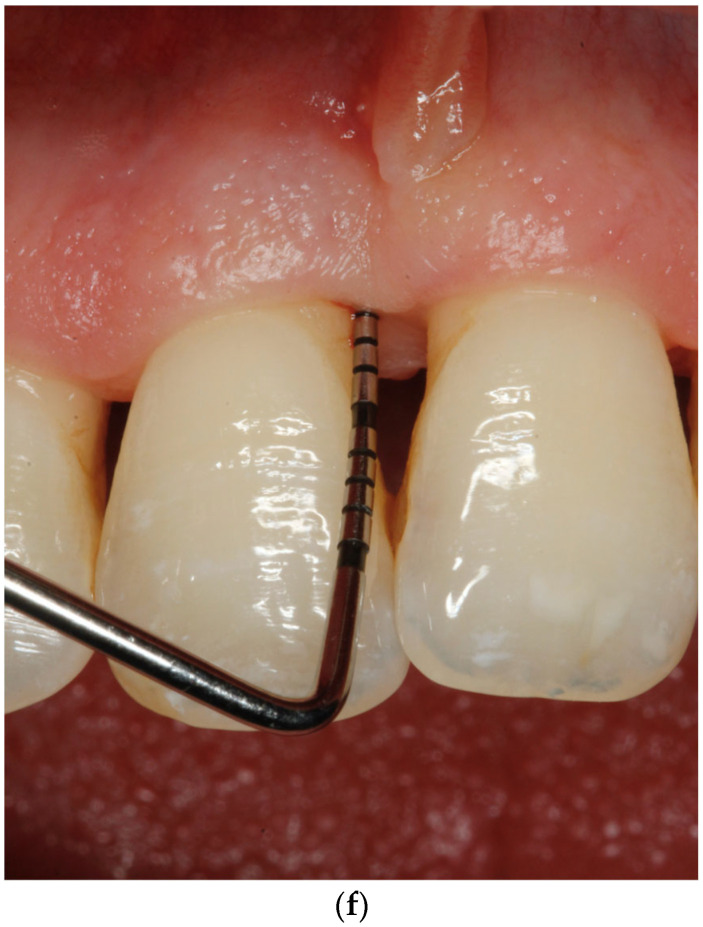
The Entire Papilla Preservation Technique (EPPT). All photographs were taken at the Department of Periodontology and Oral Mucosa Diseases, Medical University of Warsaw. (**a**). Preoperative situation. Probing pocket depth (PPD) at the mesial aspect of tooth 11: 6 mm. (**b**) A vertical incision located on the distal aspect of tooth 11, extending slightly beyond the mucogingival junction. (**c**) A full-thickness buccal flap was elevated using a micro-elevator introduced through the vertical incision until several millimeters of the preserved buccal cortical bone were exposed. In this technique, the periosteum is not incised. (**d**) Inflammatory granulation tissue was removed, and the root surface of tooth 11 was debrided and planed. A complex intrabony defect was observed on the mesial aspect, with a coronal horizontal component and a narrow two-wall apical component (5 mm) associated with labial bone dehiscence. The root surface was conditioned with 24% EDTA (PrefGel, Straumann, Basel, Switzerland) for 2 min, rinsed with saline, and treated with Emdogain (Straumann, Basel, Switzerland). The defect and dehiscence area were filled with an allogeneic bone graft mixed with Emdogain. (**e**) The flap was repositioned and stabilized in its original position without tension. The margins of the vertical incision were closed with interrupted sutures (Seralon 7/0) (Serag-Wiessner GmbH & Co. KG, Naila, Germany). An additional sling suture was placed around tooth 21 (Seralon 6/0). (**f**) Clinical situation after 12 months. Esthetics remained stable, and the probing pocket depth (PPD) at the mesial aspect of tooth 11 was 2 mm.

Consequently, surgical practices have begun transitioning toward approaches that are more tolerable for the patient, adopting the concept of minimally invasive procedures, which include restricting the size and extension of the surgical flap to decrease tissue trauma and improve the stability of the vital blood clot necessary for healing [31]. New surgical methods emerged, such as the Single Flap Approach and the Modified Minimally Invasive Surgical Technique (M-MIST) [32,33,34,35,36]. Papilla Preservation Techniques (PPTs) maintain the integrity of the interdental papilla during periodontal regenerative surgery. Unlike traditional flaps, PPTs and The Entire Papilla Preservation Technique (EPPT) include the entire papilla, minimizing wound instability that can impair regeneration [37,38,39,40,41,42]. Therefore, it is essential to understand that maintaining regeneration space is critical, as impairment of the wound healing system deteriorates desired regeneration outcomes [6,19] (Figure 1).

### 1.3. A Paradigm Shift in Defect Management: NSPT and Changes in Osseous Morphology

Subgingival non-surgical periodontal therapy (NSPT) represents step 2 of periodontal treatment, targeting disruption of the subgingival microbial biofilm and the resolution or reduction in inflammation in the supracrestal periodontal tissues. Numerous original studies and reviews published over the past decades have emphasized its critical role in periodontal therapy and long-term tooth survival [14]. In intrabony defects, NSPT is commonly regarded as an essential step to reduce inflammation and prepare tissues for surgical regeneration. Based on recent evidence, the authors advocate for a paradigm shift in the treatment of intrabony periodontal defects, highlighting that Non-Surgical Periodontal Therapy (NSPT) plays a more significant role than previously recognized, extending its potential beyond mere preparation for surgery [43]. New evidence shows that NSPT, particularly Minimally Invasive Non-Surgical Periodontal Therapy (MINST), results in radiographic defect reduction (bone gain) in intrabony defects, often ranging from 1.5 mm to approximately 3 mm up to 12 months post-treatment. Radiographic evidence suggests changes in osseous morphology can occur following NSPT, challenging older concepts that expected minimal bone changes [41]. Studies have investigated minimally invasive non-surgical approaches for managing deep intrabony defects.

These observations have stimulated interest in refining non-surgical approaches to maximize debridement efficacy while preserving soft tissues. In this context, endoscopic-assisted subgingival debridement (EASD) has emerged as an advanced minimally invasive modality, aiming to enhance visualization and precision during NSPT, particularly in deep intrabony defects.

### 1.4. Endoscopic-Assisted Subgingival Debridement (EASD)

Advances in imaging have enabled endoscopic-assisted subgingival debridement, which enhances debridement effectiveness and may improve clinical outcomes in both steps 1 and 2 of periodontal therapy. A periodontal endoscope is a specialized dental imaging device used to visualize the subgingival environment during periodontal therapy, providing real-time, magnified, and illuminated views of root surfaces, periodontal pockets, and defects that are otherwise impossible to see directly. The core of the system is a tiny camera attached to a flexible fiberoptic probe, typically less than 1 mm in diameter, which delivers powerful illumination and transmits a real-time, highly magnified image—often between 24× and 48×, and in some systems up to 100×—to a chairside monitor.

Evidence on the advantages of periodontal endoscope-assisted subgingival debridement (EASD) is inconsistent. While some studies show no clear clinical improvement, others report enhanced outcomes—such as greater reductions in pocket depth, increased attachment gain, and less residual calculus, especially in deeper sites. Heterogeneity in study protocols, defect anatomy, and clinical conditions limit firm conclusions [44,45,46,47,48,49,50,51,52,53,54,55,56]. The role of periodontal endoscopy in routine care continues to be investigated. Emerging evidence indicates that, with appropriate case selection, the adjunctive use of an endoscope may facilitate more thorough debridement and contribute to improved long-term clinical outcomes [57].

Directly comparing EASD with papilla-preservation surgery is clinically important because, while papilla-preservation techniques are effective, they are invasive, can cause postoperative morbidity, and may compromise soft tissue integrity. EASD, as a minimally invasive approach, has the potential to achieve similar clinical outcomes while preserving the interdental papilla, reducing patient discomfort, and maintaining a stable wound environment. Evaluating its effectiveness against established regenerative procedures can therefore clarify its clinical role, inform decision-making, and guide future research in less invasive periodontal therapies.

By critically examining the existing literature, this review seeks to determine whether endoscope-enhanced nonsurgical therapy can provide outcomes comparable to or supportive of established regenerative surgical approaches.

## 2. Materials and Methods

A narrative review approach was chosen due to the substantial heterogeneity of the available evidence and the limited number of high-quality comparative studies directly addressing periodontal endoscopy-assisted therapy in intrabony defects. The paucity of randomized trials and the absence of sufficiently homogeneous data precluded the performance of a systematic review or meta-analysis.

An electronic search of the literature was performed with a stop date of November 2025 to identify studies comparing periodontal endoscopy-assisted nonsurgical periodontal therapy with surgical regenerative procedures, including papilla-preservation techniques (EPPT, M-PPT, SPPT) for the management of intrabony periodontal defects. Four databases—PubMed/MEDLINE, Web of Science, Scopus, and the Cochrane Library—were systematically screened. The search retrieved a total of 1037 records, including 259 from PubMed, 38 from Web of Science, 733 from Scopus, and 7 from the Cochrane Library.

Prior to formal screening, the first 50 titles and abstracts were used to calibrate the two independent reviewers with oversight from an experienced supervising researcher. Agreement between reviewers was assessed using the kappa statistic. Following calibration, two reviewers independently screened all titles and abstracts. Full texts of potentially relevant studies were retrieved for eligibility assessment, and any disagreements were resolved through discussion with the supervising researcher.

All references were imported into a reference management software system, Mendeley Reference Manager (version 2.120.0; Elsevier, Amsterdam, The Netherlands), where 397 duplicates were removed, leaving 640 unique records for screening. After title and abstract screening, 580 records were excluded, and 60 studies were sought for full-text review. Of these, two studies met the inclusion criteria and were included in the final review.

**Figure 2:**
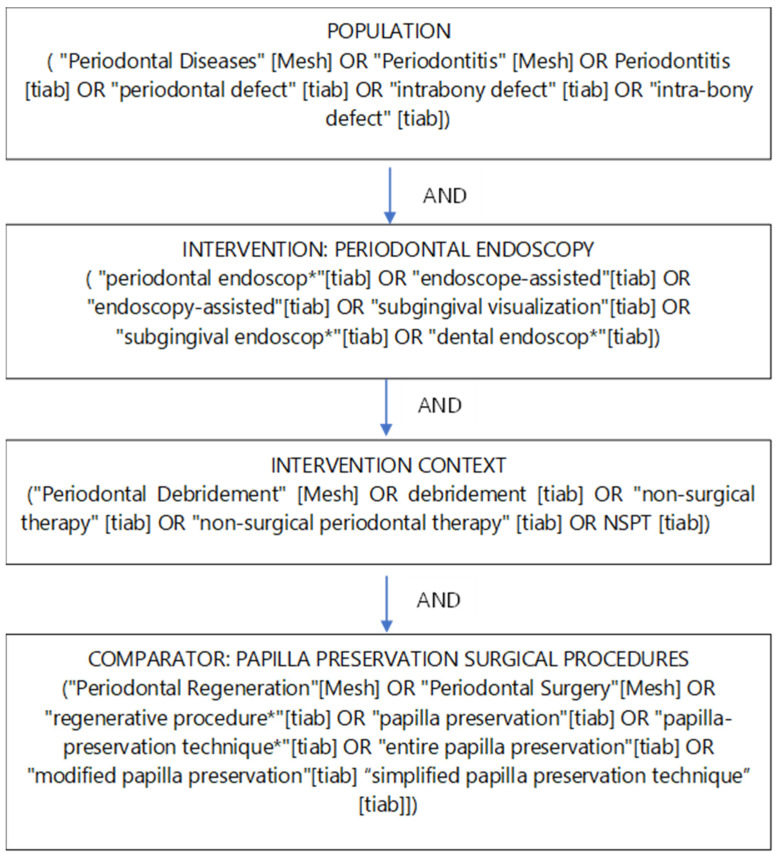
Boolean Diagram (Concept Map of Search Strategy.

The search strategy was constructed using MeSH terms and free-text keywords combined with Boolean operators. The conceptual structure followed the Population–Intervention–Comparison (PIC) framework. These elements were combined using AND/OR operators as illustrated in the Boolean diagram (Concept Map of Search Strategy) provided in the source material (Figure 2).

**Figure 3:**
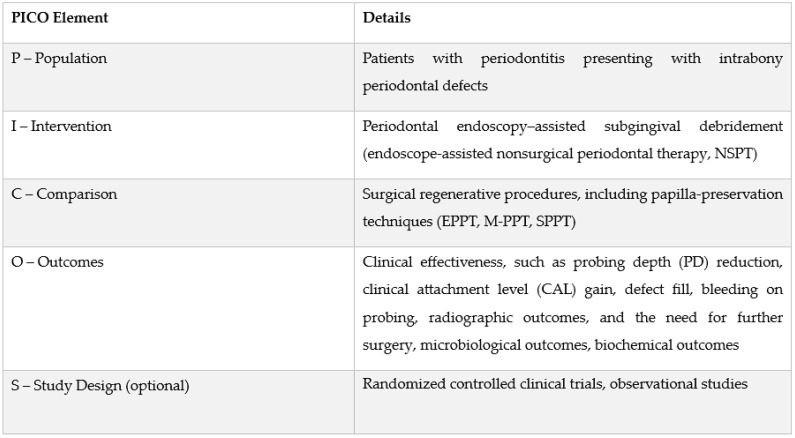
PICOS framework.

The selection criteria were structured addressing the following PICOS question: “*What is the effectiveness of periodontal endoscopy-assisted subgingival debridement compared with surgical regenerative procedures (including papilla-preservation techniques) in the treatment of intrabony periodontal defects?*” (Figure 3).

Studies were included if they met the following eligibility criteria: (1) human patients diagnosed with periodontitis presenting intrabony defects; (2) interventions involving periodontal endoscopy-assisted subgingival debridement; (3) comparison with surgical regenerative procedures employing papilla-preservation techniques (EPPT, M-PPT, SPPT); (4) reported clinical, radiographic, microbiological, or biochemical outcomes; (5) study designs included randomized controlled trials or observational studies.

Studies were excluded if they involved animal models, in vitro experiments, lacked a comparison with surgical regenerative therapy, did not report relevant outcomes, or were not published in English.

**Figure 4:**
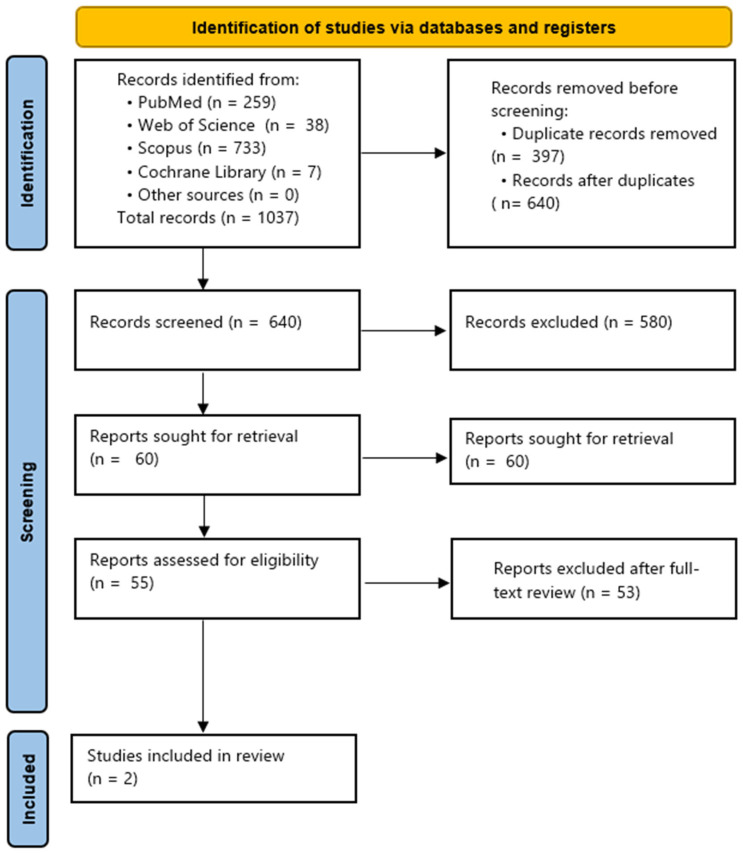
A PRISMA-style flow diagram.

From the studies meeting the inclusion criteria, relevant data were extracted and organized into evidence tables. A PRISMA-style flow diagram (Figure 4) was generated to visualize the study selection process. The results were synthesized narratively due to the limited number of included studies (*n* = 2) and heterogeneity in study protocols and outcome measures. Data from the selected studies were extracted and organized into tables.

## 3. Results

A total of 1037 articles were retrieved from the database searches, including PubMed, Web of Science, Scopus, and Cochrane Library. The final set of 2 studies [5,18] focusing on the effectiveness of periodontal endoscopy-assisted subgingival debridement (EASD) compared with surgical regenerative procedures (including papilla-preservation techniques) in the treatment of intrabony periodontal defects were included in the review. The main objectives of the chosen studies and a summary of reported results are depicted in Table 1 and Table 2. Included reports were finally discussed.

### 3.1. Description of Included Studies

The two selected reports [5,18] originated from the same research group and patient cohort. One study was a randomized controlled non-inferiority trial [5] evaluating the clinical and patient-reported outcomes of endoscope-assisted subgingival debridement (EASD) versus papilla preservation flap surgery (PPFS) in patients with residual intrabony defects over a 12-month follow-up. The second study [18] was a prospective observational analysis focusing on subgingival microbiological changes within the same treated sites, using longitudinal sampling and 16S rRNA sequencing to assess microbial community composition and stability following EASD or PPFS. The clinical randomized controlled trial [5] included 62 patients (30 treated with EASD and 32 with PPFS) with a 12-month follow-up, while the accompanying microbiological study [18] analyzed a subgroup of 19 patients (PPFS, *n* = 11; EASD, *n* = 8) with longitudinal subgingival sampling at multiple time points over 12 months.

### 3.2. Clinical and Procedural Outcomes: Non-Inferiority of EASD

The study by Ho et al. (2025) [5] demonstrated that EASD was non-inferior to PPFS regarding the primary outcome, which was the change in Clinical Attachment Level (CAL) at 12 months. The mean CAL gain was 2.0 ± 1.0 mm for the EASD group and 1.8 ± 1.0 mm for the PPFS group. The 95% Confidence Interval (CI) of the inter-group difference was −0.3 to 0.8 mm, which was within the stipulated 1 mm non-inferiority margin. Furthermore, secondary efficacy outcomes, including pocket resolution (defined as no PPD > 5 mm and no PPD > 4 mm with BOP+) achieved in over 87% of cases for both treatments at all time points and radiographic bone changes (including bone fill and crest position stability), showed no significant inter-group differences. Notably, EASD offered procedural advantages: the treatment time was significantly shorter for EASD (14 ± 4.8 min) compared to PPFS (20.6 ± 5.8 min) (*p* < 0.01). The minimally invasive nature of EASD also resulted in significantly better early wound healing index (EWHI) scores at all time points.

### 3.3. Subgingival Microbial Dynamics: Stability vs. Variability

The prospective study by Ho et al. (2025) [18] investigated changes in the subgingival microbial community following two distinct treatments for residual periodontal intrabony defects requiring Step 3 therapy. Patients with periodontitis were randomly assigned to PPFS or EASD and subgingival plaque samples were collected over 12 months. The primary aim was to understand the subgingival microbial changes induced by these two methods.

According to the authors, subgingival microbiome analysis showed largely comparable microbial diversity and community composition between the PPFS and EASD groups throughout the study.

The authors report that time-series analysis revealed that both PPFS and EASD substantially reduced subgingival microbial load shortly after treatment, as reflected by decreased alpha diversity at day 3. However, their immediate effects differed: PPFS caused a pronounced shift in microbial community structure compared with baseline, whereas EASD induced more modest, non-significant changes.

The relative levels of both disease-associated and health-associated taxa remained largely comparable between the PPFS and EASD groups over time. Following treatment, periodontopathogenic bacteria showed a marked reduction in both groups, with the greatest decrease observed shortly after surgery, followed by partial recolonization within one month. Beneficial periodontal species were also reduced after intervention, indicating that both treatments broadly influenced the subgingival microbial ecosystem.

The EASD group demonstrated greater microbial stability over time, with no significant shifts in community structure across follow-up periods (the 12-month microbial profile closely resembled baseline conditions). In contrast, the PPFS group showed more pronounced temporal changes (with microbial composition at 12 months remaining distinct from the initial state, indicating lower long-term community stability).

## 4. Discussion

The decision to conduct a narrative review reflects the current state of the evidence, which is characterized by methodological heterogeneity, small study populations, and a lack of direct, high-quality comparisons with established regenerative surgical approaches. In this context, a narrative synthesis allows for a critical and conceptual discussion of emerging therapeutic strategies while explicitly acknowledging existing evidence gaps.

This narrative review identified a very limited but focused body of evidence directly comparing periodontal endoscopy-assisted subgingival debridement (EASD) with surgical regenerative procedures incorporating papilla-preservation techniques for the treatment of intrabony periodontal defects. However, the overall level of evidence remains limited as relatively few well-designed clinical studies have directly evaluated periodontal endoscopy-assisted therapy in intrabony defect treatment, particularly in comparison with established regenerative surgical approaches. In contrast, regenerative periodontal surgery—especially when performed using papilla-preservation techniques—has been extensively investigated and is supported by a broader body of evidence demonstrating predictable clinical attachment gain and radiographic defect fill in appropriately selected cases.

From over one thousand screened records, only two eligible studies were included [5,18], both originating from the same research group. Together, these investigations provide complementary clinical and microbiological perspectives on the comparative effectiveness of EASD and papilla preservation flap surgery (PPFS) in Step 3 periodontal therapy. Clinically, EASD was demonstrated to be non-inferior to PPFS in terms of clinical attachment level gain, pocket resolution, radiographic bone stability, and patient-reported outcomes over a 12-month follow-up, while offering procedural advantages such as reduced treatment time and improved early wound healing. Microbiologically, both interventions produced comparable reductions in periodontal pathogens and similar overall community composition; however, EASD was associated with greater long-term stability of the subgingival microbiome, whereas PPFS induced more pronounced and persistent shifts in microbial structure. Collectively, these findings suggest that EASD may represent a minimally invasive alternative to papilla-preservation regenerative surgery in selected intrabony defects, although the strength of this conclusion is constrained by the small number of available studies and their shared cohort.

In the evolving landscape of Step 3 periodontal therapy, a recent systematic review and meta-analysis by Ho et al. [57] has provided a high-level synthesis of the efficacy of Endoscope-Assisted Subgingival Debridement (EASD). This comprehensive analysis scrutinized five randomized controlled trials (RCTs) involving 155 subjects and a total of 4072 sites to determine if the adjunctive use of a periodontal endoscope offers superior clinical benefits for managing residual pockets.

When compared against the conventional approach of Repeated Root Surface Debridement (RSD), the results were definitive [58,59,60]. The review demonstrated that EASD leads to significantly enhanced clinical outcomes. Specifically, at the 6-month post-operative mark, sites treated with endoscopic assistance achieved a weighted mean difference of 0.84 mm greater reduction in Probing Pocket Depth (PPD) and 0.89 mm greater gain in Clinical Attachment Level (CAL) compared to those receiving standard repeated instrumentation. Furthermore, EASD showed a 20% higher likelihood of achieving complete pocket resolution (<4 mm).

Perhaps most significant for clinical decision-making is the comparison between EASD and traditional Access Flap Periodontal Surgery (AFPS) [5,61,62,63]. The meta-analysis revealed no significant differences in CAL gain, PPD reduction, or the prevalence of pocket resolution between these two modalities. This finding is a cornerstone for the “paradigm shift” in periodontal care, as it suggests that EASD is a statistically non-inferior, viable non-surgical alternative to invasive surgical access for certain deep periodontal defects [57].

Although direct comparisons with regenerative surgical approaches remain limited, increasing evidence indicates that minimally invasive strategies, including papilla-preservation surgical techniques and minimally invasive non-surgical therapy (MINST) [58,59], can achieve favorable periodontal healing in intrabony defects even without the use of regenerative materials. Studies have shown that MINST can promote meaningful clinical improvement in deep periodontal pockets, supporting the concept that careful, atraumatic root surface debridement is a critical determinant of healing. Within this paradigm, periodontal endoscopy-assisted subgingival debridement may be considered an advanced or “enhanced” form of MINST, offering improved visualization while preserving soft tissues. At the same time, emerging approaches that combine regenerative biomaterials with flapless or minimally invasive debridement suggest a potential convergence between non-surgical and regenerative treatment concepts [55]. Further research is needed to define whether EASD should be positioned primarily as an optimized MINST modality or as an adjunct facilitating minimally invasive periodontal regeneration.

Defect morphology represents a critical determinant of treatment outcomes in intrabony periodontal defects. As highlighted by Nibali et al. [22], a wide range of tooth types and local anatomical variables—including single- versus multi-rooted teeth, furcation involvement, furcation trunk length, and the dimensions of intrabony defects—may significantly influence the response to different therapeutic modalities. Moreover, factors such as defect configuration (one-, two-, or three-wall defects), defect depth and width, and the degree of anatomical containment play a pivotal role in determining clot stability, wound healing, and the potential for periodontal regeneration. These morphological characteristics should therefore be carefully considered when selecting between minimally invasive non-surgical approaches, such as periodontal endoscopy-assisted subgingival debridement, and surgical regenerative procedures incorporating papilla-preservation techniques.

The clinical potential of Endoscope-Assisted Subgingival Debridement (EASD) has recently evolved from a purely debridement-focused tool into a versatile platform for Non-Incisional Regeneration (NIT). As demonstrated by Shi et al. [55], NIT describes a flapless, endoscopy-guided approach that enables placement of regenerative biomaterials into intrabony defects through the periodontal pocket, without papilla incision or flap elevation. Using high-magnification visualization and minimal soft-tissue manipulation, this approach aims to preserve vascular supply and blood clot stability—key prerequisites for periodontal regeneration.

Preliminary data from proof-of-concept studies suggest that NIT may achieve clinically meaningful improvements in probing depth and attachment levels compared with debridement alone. However, these findings are derived from limited, non-comparative studies and have not been directly evaluated against established papilla-preservation surgical techniques. Therefore, NIT should currently be regarded as an emerging, hypothesis-generating application of endoscopy-assisted therapy rather than a validated alternative to surgical regeneration. Consequently, NIT is discussed in this review solely to illustrate a potential future direction of minimally invasive periodontal regeneration and to highlight an unresolved knowledge gap, not to suggest clinical interchangeability with surgical regeneration. Further controlled trials are required to define its indications, reproducibility, and comparative effectiveness.

Endoscope-assisted subgingival debridement (EASD) may be particularly suitable for carefully selected patients and defect morphologies where a minimally invasive approach is desirable. Potential clinical indications include residual intrabony defects following adequate non-surgical therapy, especially in esthetically sensitive areas where preservation of the interdental papilla and avoidance of gingival recession are critical. EASD may also be considered in patients with limited tolerance for surgical procedures, increased morbidity risk, or a preference for nonsurgical interventions. Additionally, defects with contained morphology and sufficient access through the periodontal pocket may be more amenable to endoscopy-assisted treatment. However, advanced, wide, or non-contained defects requiring space maintenance and extensive regeneration may still benefit more predictably from surgical papilla-preservation techniques. Careful case selection remains essential, and current evidence does not support replacing surgical regeneration in all clinical scenarios.

## 5. Limitations

Several limitations must be acknowledged when interpreting the findings of this narrative review and the underlying evidence.

First, the overall body of literature directly comparing periodontal endoscopy-assisted subgingival debridement (EASD) with surgical regenerative procedures incorporating papilla-preservation techniques (PPFS) is very limited. Only two primary studies met the inclusion criteria, both originating from the same research group and evaluating the same patient cohort, which restricts the generalizability of the results and increases the risk of bias. This small and homogeneous sample may not fully represent the broader population of patients with intrabony defects.

Second, the available studies had relatively short follow-up periods, with the longest being 12 months. While these data provide preliminary insights into clinical and microbiological outcomes, longer-term follow-up is essential to evaluate the durability of clinical attachment gains, radiographic defect fill, and microbiome stability over time. The lack of extended observation limits the ability to draw firm conclusions about the long-term efficacy of EASD relative to papilla-preservation regenerative surgery.

Third, most studies relied primarily on clinical and radiographic assessments to evaluate treatment outcomes. Histological confirmation of true periodontal regeneration—i.e., new formation of cementum, periodontal ligament, and alveolar bone—was not performed, which constrains understanding of the biological mechanisms underlying observed clinical improvements. Without histological data, it is difficult to determine whether EASD achieves true regeneration or merely wound healing with connective tissue attachment.

Fourth, defect morphology and anatomical variability represent important confounding factors that were not fully controlled. Tooth type, number of remaining bony walls, defect depth and width, furcation involvement, and local anatomical considerations can significantly influence treatment outcomes. Most reviewed studies did not stratify results according to these morphological variables, limiting the ability to identify which defect types are most suitable for EASD versus surgical regenerative approaches.

Fifth, patient-related factors such as age, systemic health, oral hygiene, and smoking status were either inconsistently reported or controlled across studies, potentially influencing clinical outcomes. Moreover, variations in operator skill, instrumentation, and surgical technique could also affect results, particularly given the technical sensitivity of endoscopic and papilla-preservation procedures.

Taken together, these limitations underscore the need for further high-quality, multi-center, randomized controlled trials with larger sample sizes, extended follow-up, standardized defect classifications, and incorporation of histological evaluation. Such studies are essential to clarify the comparative effectiveness of EASD, papilla-preservation regenerative surgery, and emerging minimally invasive regenerative approaches in the management of intrabony periodontal defects.

## 6. Conclusions

Given the limited and heterogeneous nature of the available evidence, this narrative review should be regarded as a hypothesis-generating overview rather than a definitive comparative assessment. Periodontal endoscopy-assisted therapy may be considered a promising minimally invasive approach for selected intrabony defects, potentially reducing surgical morbidity and preserving interdental tissues. Although early data suggest that endoscopy-guided approaches may offer comparable clinical improvements with less invasiveness, the evidence base is too small to support definitive recommendations. Robust, well-designed randomized trials are needed to define its clinical indications and compare it directly with established regenerative procedures.

## Figures and Tables

**Table 1:** Clinical and Procedural Outcomes.

Outcome Category	Parameter (Outcome)	EASD Result (Test)	PPFS Result (Control)	Inter-Group Comparison (*p*-Value/Conclusion)
I. Primary	CAL Gain (Change at 12 months) Mean ± SD (95% CI)	2.0 ± 1.0 mm	1.8 ± 1.0 mm	Non-inferiority demonstrated. Inter-group difference 0.3 (95% CI: −0.3 to 0.8 mm) (within 1 mm margin)/*p* = 0.346
II. Secondary	Pocket Resolution. No PPD > 4 mm with bleeding, *n* (%)/No deep residual pocket PPD > 5 mm, *n* (%)	>87% of sites	>87% of sites	No significant inter- group differences at any time point (*p* > 0.343)
	Radiographic Bone Changes (Bone fill, INTRA)	Bone gain: 1.7 ± 1.5 Depth of the intrabony component (INTRA): 1.5 ± 1.4	Bone gain: 1.2 ± 1.1 Depth of the intrabony component (INTRA): 1.3 ± 1	No significant inter- group differences were observed. (*p* = 0.191 for bone gain) (*p* = 0.444 for intrabony defect)
	Treatment Time	14 ± 4.8 min	20.6 ± 5.8 min	EASD significantly shorter (*p* < 0.01).
	Early Wound Healing Index (EWHI)	Significantly better scores at all time points	Lower scores at all time points	EASD significantly better.
	Pain Level (VAS) (during procedure) (VAS scale, with no pain at 0 and unbearable pain at 100 mm)	6.5 ± 11.9	5.0 ± 8.9 mm	The inter- group difference was not significant (*p* = 0.574)
	Procedure Difficulty (VAS) (VAS scale: very easy = 0, very difficult = 100)	15.2 ± 16.4 mm	15.7 ± 18.1 mm	The inter- group difference was not significant (*p* = 0.918)

**Table 2:** Subgingival Microbial Dynamics Over Time.

1. Baseline Microbial Composition	2. Immediate Post-Treatment Shifts	3. Longitudinal Microbial Changes	4. Community Stability & Function	5. Microbe–Clinical Correlations
Comparable alpha and beta diversity between PPFS and EASD at baseline and all follow-up points	Both treatments reduced microbial load at day 3	Dominant taxa consistently represented >50% of the community	EASD showed minimal temporal variation in community structure	Commensal taxa negatively correlated with Pl, BOP, and PPD
	PPFS induced a significant community shift vs. baseline	Disease- and health-associated taxa showed similar relative abundance between groups	No significant community shifts in EASD over time	Pathogenic taxa positively correlated with clinical parameters
	EASD showed modest, non-significant microbiome changes	Periodontopathogens markedly reduced post-treatment	EASD 12-month microbiome closely resembled baseline	BOP associated with *Fusobacterium nucleatum* subsp. *vincentii*
	Shared and unique taxa numbers comparable between PPFS and EASD	Partial recolonization of pathogens observed by 1 month	PPFS microbiome remained distinct from baseline at 12 months	PPD associated with *Fretibacterium fastidiosum* and *Treponema socranskii*

## Data Availability

No new data were created or analyzed in this study. Data sharing is not applicable to this article.

## Abbreviations

The following abbreviations are used in this manuscript:

AFPS	Access Flap Periodontal Surgery
BOP	Bleeding on Probing
CAL	Clinical Attachment Level
CI	Confidence Interval
EASD	Endoscope-Assisted Subgingival Debridement
EPPT	Entire Papilla Preservation Technique
EWHI	Early Wound Healing Index
FMBS	Full Mouth Bleeding Score
FMPS	Full Mouth Plaque Score
GTR	Guided Tissue Regeneration
INTRA	Intrabony Component (context: depth of the intrabony component)
ITT	Intention-To-Treat
M-MIST	Modified Minimally Invasive Surgical Technique
MINST	Minimally Invasive Non-Surgical Periodontal Therapy
M-PPT	Modified Papilla Preservation Technique
NIT	Non-Incisional Regeneration Technique
NSPT	Non-Surgical Periodontal Therapy
PICOS	Population, Intervention, Comparison, Outcomes, Study Design
PPD	Probing Pocket Depth
PPFS	Papilla Preservation Flap Surgery
PPTs	Papilla Preservation Techniques
PRISMA	Preferred Reporting Items for Systematic Reviews and Meta-Analyses (style of flow diagram)
PSRP	Periodontal Scaling and Root Planing
RCT	Randomized Controlled Trial
RSD	Root Surface Debridement
SPPT	Simplified Papilla Preservation Technique
VAS	Visual Analogue Scale
